# PIKAChU: a Python-based informatics kit for analysing chemical units

**DOI:** 10.1186/s13321-022-00616-5

**Published:** 2022-06-07

**Authors:** Barbara R. Terlouw, Sophie P. J. M. Vromans, Marnix H. Medema

**Affiliations:** Bioinformatics Group, Wageningen University, Droevendaalsesteeg 1, 6708 PB Wageningen, The Netherlands

**Keywords:** Cheminformatics kit, Python, Structure visualisation, In silico chemistry, Molecular fingerprinting

## Abstract

As efforts to computationally describe and simulate the biochemical world become more commonplace, computer programs that are capable of in silico chemistry play an increasingly important role in biochemical research. While such programs exist, they are often dependency-heavy, difficult to navigate, or not written in Python, the programming language of choice for bioinformaticians. Here, we introduce PIKAChU (Python-based Informatics Kit for Analysing CHemical Units): a cheminformatics toolbox with few dependencies implemented in Python. PIKAChU builds comprehensive molecular graphs from SMILES strings, which allow for easy downstream analysis and visualisation of molecules. While the molecular graphs PIKAChU generates are extensive, storing and inferring information on aromaticity, chirality, charge, hybridisation and electron orbitals, PIKAChU limits itself to applications that will be sufficient for most casual users and downstream Python-based tools and databases, such as Morgan fingerprinting, similarity scoring, substructure matching and customisable visualisation. In addition, it comes with a set of functions that assists in the easy implementation of reaction mechanisms. Its minimalistic design makes PIKAChU straightforward to use and install, in stark contrast to many existing toolkits, which are more difficult to navigate and come with a plethora of dependencies that may cause compatibility issues with downstream tools. As such, PIKAChU provides an alternative for researchers for whom basic cheminformatic processing suffices, and can be easily integrated into downstream bioinformatics and cheminformatics tools. PIKAChU is available at https://github.com/BTheDragonMaster/pikachu.

**Graphical Abstract:**

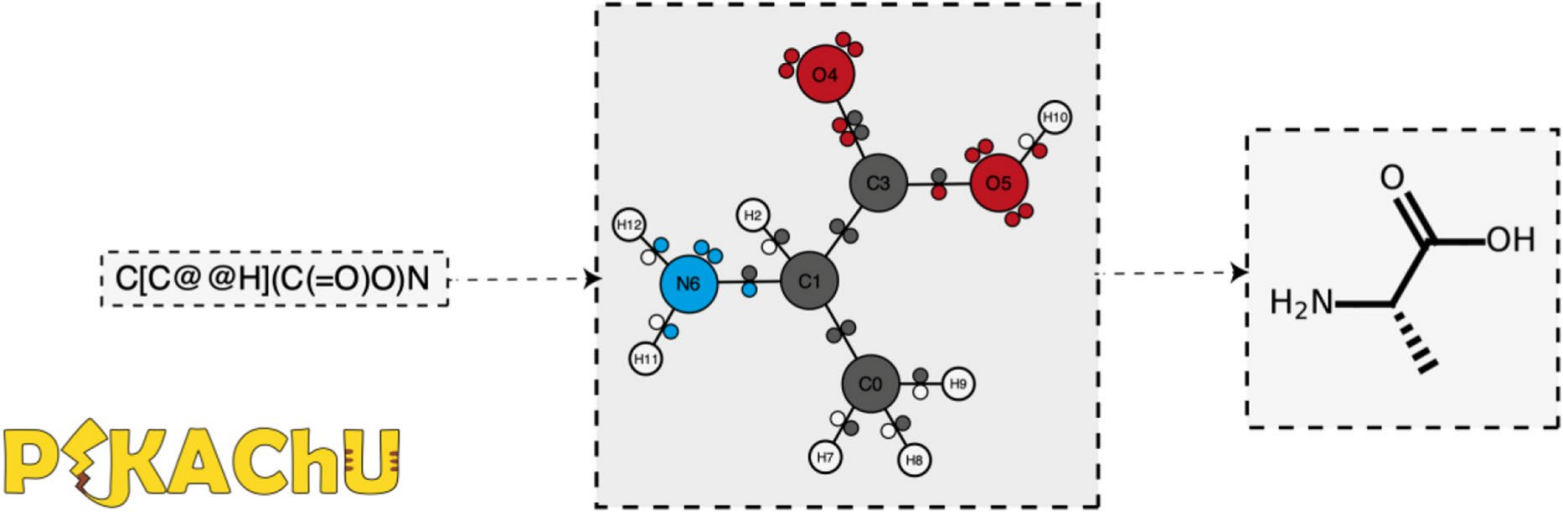

**Supplementary Information:**

The online version contains supplementary material available at 10.1186/s13321-022-00616-5.

## Introduction

In a data-driven world where the discovery of novel natural and synthetic molecules is increasingly necessary, in silico chemical processing has become an essential part of biological and chemical research. Novel metabolites are compared or added to searchable chemical databases such as ChEBI [6], PubChem [10], NP Atlas [20], and COCONUT [17]; molecular structures are predicted from biological pathways [3, 16]; and bioactivities and pharmaceutical properties are predicted from chemical structure [1, 18, 21]. Such analyses rely on robust cheminformatics kits that can perform basic chemical processing, such as fingerprint-based similarity searches, substructure matching, molecule visualisation and chemical featurisation for machine learning purposes.

Typically, molecular processing by cheminformatics kits begins with the reading in of molecular data from chemical data formats, ranging from one-dimensional to three-dimensional molecular representations. One such formats is the SMILES (Simplified Molecular-Input Line Entry System) format, which represents a molecule as a one-dimensional string, describing atom composition, connectivity, stereochemistry, and charge. More elaborate formats such as PDB and MOL use text files to store not just the abovementioned properties but also atom coordinates in three-dimensional space.

Depending on the application, different formats and subsequent processing are appropriate. Due to the vast number of possible chemical analyses, exhaustive cheminformatics kits have accumulated into software libraries that are so large that they can be hard to navigate, and rely on so many dependencies that they can be difficult to implement in software packages. As a result, the trade-off between time spent accessing and integrating these cheminformatics kits into a codebase and time spent on actual analyses is disproportionate for users that need to perform simple in silico analyses such as reading in SMILES, drawing a molecule, or visualising a substructure. One popular open-source cheminformatics kit that suffers from this problem is RDKit [11]. While RDKit is an incredibly fast and powerful library that supports an immense variety of possible chemical operations, its use of both Python and C++ as programming languages as well as the sheer number of dependencies it relies on frequently causes compatibility issues when integrating RDKit into other programs, and disproportionately increases the number of libraries that need to be installed. Therefore, while RDKit is great for heavy-duty in silico analyses such as computing 3D conformers for a compound or constructing electron density maps, it is a bit heavyweight for the basic operations that most researchers in bioinformatics and cheminformatics require.

A second widely-used cheminformatics kit is CDK [22]. Written in Java, it is well-suited for implementation in web applications, and has successfully been used for molecular processing in the COCONUT database [17], the Cytoscape application chemViz2 [13], and the scientific workflow platform KNIME (Konstanz Information Miner) [2]. However, with Python becoming the programming language of choice for many scientists [4], especially those working in the growing field of (deep) neural networks, CDK is not always an ideal fit.

To make basic cheminformatics processing more accessible for Python programmers, we therefore introduce PIKAChU: a Python-based Informatics Kit for Analysing Chemical Units. PIKAChU is a flexible cheminformatics tool with few dependencies. It can parse molecules from SMILES, visualise chemical structures and substructures in matplotlib, perform Extended Connectivity FingerPrinting (ECFP) [15] and Tanimoto similarity searches, and execute basic reactions with a focus on natural product chemistry. Therefore, we hope that PIKAChU can provide a convenient alternative for many Python-based bio- and cheminformatics tools and databases that only demand basic chemical processing.

## Methods and implementation

### Software description

PIKAChU is implemented in Python (v3.9.7). Its only dependency is the common Python package matplotlib (v3.4.3). PIKAChU can be run on Windows, MacOS, and Linux systems.

### Parsing molecules from SMILES

**Fig. 1:**
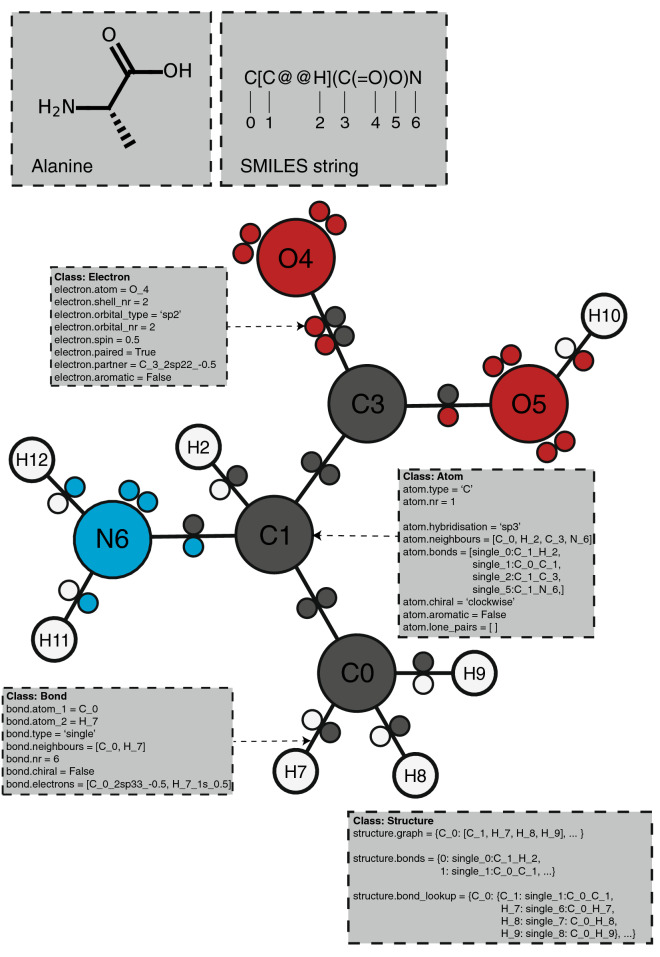
Overview of the internal structure of PIKAChU's structure graphs. This example uses L-alanine, a small amino acid. The four bottom boxes in grey indicate attributes for each of PIKAChU's major classes: Structure, Atom, Bond and Electron.

PIKAChU takes a SMILES string as input and from it builds a graph object, in which nodes represent atoms and edges represent bonds (Fig. 1). For each atom, PIKAChU initially stores information on chirality, aromaticity, charge, and connectivity. For each bond, it stores bond type (single, double, triple, quadruple, or aromatic), neighbouring atoms, and cis–trans stereochemistry for double bonds. Once all atoms, bonds, and their connectivities have been stored, electron shells and orbitals are constructed for each non-hydrogen atom. Next, we determine the valency for each non-hydrogen atom, taking into account atom charge. For atoms of variable valency such as sulphur (2, 4 or 6) and phosphorus (3 or 5), we select a valency that is equal to or higher than the sum of non-hydrogen bonds and explicit hydrogen bonds, prioritising smaller valencies. Double, triple, quadruple and aromatic bonds contribute proportionally to this sum. If insufficient bonding orbitals are available to achieve the desired valency, the electrons in the valence shell are excited to higher-energy orbitals, such that each orbital contains at most one electron. Implicit hydrogens are then added to the structure such that the pre-determined valencies are obeyed.

An exception is made for nitrogens of valency 5, which are not chemically possible due to insufficient bonding orbitals but can sometimes be encountered in SMILES strings, especially in those describing compounds containing nitro groups. If such a valency 5 nitrogen is attached to at least one oxygen through a double bond, this double bond is interpreted as a single bond instead, the oxygen’s charge is set to − 1 and the nitrogen’s charge is set to 1, such that the nitrogen’s valency becomes 4 and bonding laws are obeyed.

Subsequently, electrons are allocated to the p-orbitals of π bonds in double, triple and quadruple bonds, and atom hybridisation is determined from steric number. Then, all cycles in the graph are detected using an open-source Python implementation [12] of the simple cycle detection algorithm described by D. Johnson in 1975 [8]. PIKAChU removes all cycles smaller than three atoms and identifies the smallest set of unique smallest rings (SSSR).

Next, the SSSR is used for aromaticity detection. This is done recursively: in each round, each cycle that has not yet been added to the set of aromatic cycles is evaluated with Hückel’s 4n + 2 rule on planar rings. We chose to assess aromatic cycles rather than systems as Hückel’s rule is not always reliable for cyclic systems [7]. First, the hybridisation of all atoms in the cycle is examined. All atoms must be sp2-hybridised, or sp3-hybridised with a delocalisable lone pair that can be promoted to a p-orbital. If the cycle is planar and the sum of double bonds and lone pairs is odd, the cycle is considered aromatic. Aromatic bond stretches are locally kekulised, and double bonds are subsequently counted. When a cycle is considered aromatic, bonds and atoms in the cycle are set to aromatic, and lone pairs of sp3-hybridised atoms are promoted to p-orbitals such that the new hybridisation is sp2. Recursion is needed in case double bonds in cyclic systems are defined in such a way that not all sub-cycles contain the required number of bonds to obey Hückel’s rule: when adjacent bonds are updated to aromatic, they will be counted in the next round of aromaticity detection (Additional file 2: Fig. S1). When, after an iteration, the number of aromatic cycles no longer changes, all aromatic cycles have been detected. From these cycles, PIKAChU defines aromatic systems, where aromatic cycles are considered part of an aromatic system if they share a bond with at least one other aromatic cycle in the system.

Electrons involved in σ bonds and aromatic bonds are only allocated after aromaticity detection. As electrons involved in aromatic systems are not localised to specific atoms or bonds, the p-orbitals of atoms in aromatic systems are emptied and their electrons stored in an AromaticSystem object.

Finally, any unpaired electrons are dropped back to lower-energy orbitals. A structure object is returned which can be visualised, kekulised, analysed through substructure matching and molecular fingerprinting, and altered through an assortment of built-in and custom chemical reactions.

If a SMILES string yields a structure object that is chemically incorrect due to too many or too few bonds being attached to an atom or valence shells not being filled appropriately in the case of organic atoms, PIKAChU gives a StructureError, informing the user that the parsed structure is chemically incorrect and gives a rough indication of why. Two examples of such StructureError messages are ‘*Error parsing "F/C(\Cl)*=*C(F)/Cl": Conflicting double bond stereochemistry*’ and ‘*Error parsing "CN(*=*O)*=*O": Basic bonding laws have been violated’*.

### Visualisation and kekulisation

Prior to visualisation, aromatic systems within a structure are kekulised so that aromatic systems can be represented by alternating single and double bonds. PIKAChU kekulises aromatic systems using a Python implementation [23] of Edmonds’ Blossom Algorithm for maximum matching [5]. Next, atoms are positioned using PIKAChU’s drawing software. PIKAChU’s python-based drawing algorithm was adapted and improved from SmilesDrawer [14], an open-source JavaScript library for molecular visualisation. While written in different programming languages, the algorithms underlying the drawing software of PIKAChU and SmilesDrawer are largely identical. We will briefly recap this algorithm below; more detailed descriptions of the algorithm’s elements can be found in the SmilesDrawer paper [14].

**Fig. 2:**
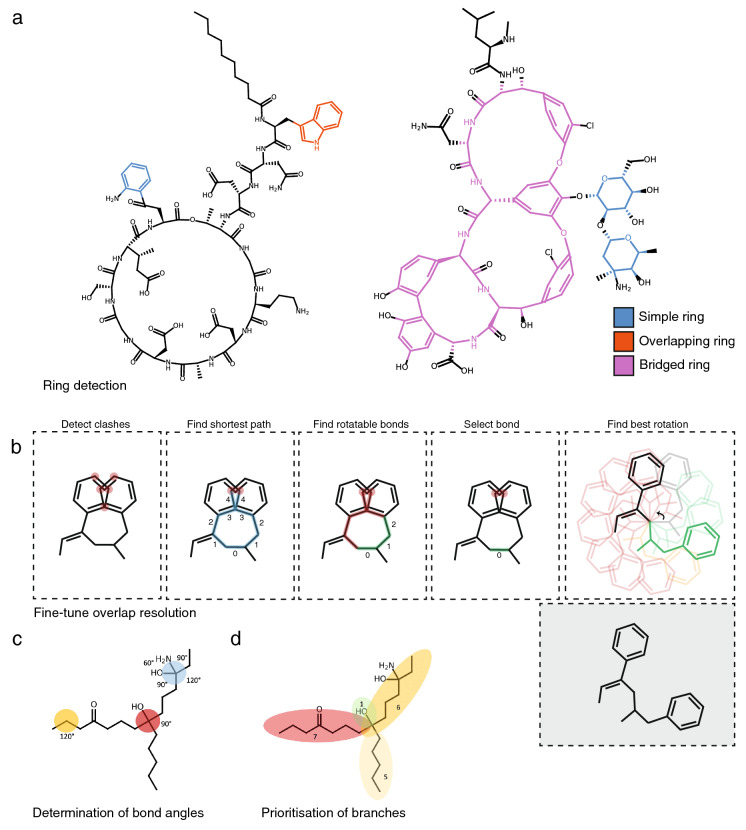
PIKAChU’s drawing algorithm. **A** Examples of simple (blue), overlapping (red) and bridged (pink) rings. Note that the aromatic rings in pink become part of the bridged ring system because they overlap with bridged rings. **B** PIKAChU’s ‘finetuning’ algorithm. First, clashes are detected and the shortest path between them is found. The rotatable bond with the shortest distance to the centre of the shortest path is chosen (indicated with numbers). 12 rotations at incremental angles of 30° are evaluated for clashes. The best rotation is chosen. **C** Determination of bond angles based on neighbouring atoms. If an atom has 3 or fewer non-hydrogen neighbours, the angles default to 120° (yellow). If an atom has 4 non-hydrogen neighbours, angles default to 90° if three or more of the branches have a depth more than 1, or three or four branches have a depth of exactly 1 (red). If however two of the branches have a depth of exactly 1 (blue), the angle is set to 120° between the two longest branches, 90° between any short branch and any long branch, and 60° between the shortest branches. **D** Positioning of neighbouring branches depends on the depth of each branch: the two longest branches (red and dark yellow, depths 7 and 6 respectively) are always placed opposite one another.

First, if indicated, PIKAChU’s drawing algorithm removes hydrogens from the graph. Next, it finds the smallest set of smallest rings in the structure graph. As SmilesDrawer’s SSSR implementation sometimes failed to detect some rings, leading to unreadable structure renderings (Additional file 2: Fig. S2), we implemented the SSSR algorithm ourselves. Next, like SmilesDrawer, PIKAChU classifies all rings into one of three groups: simple rings, overlapping rings, and bridged rings. Simple rings are standalone rings that do not have any overlapping atoms with any other rings. Overlapping rings are rings that overlap with one or more other rings, where the overlap between any two rings can comprise at most two atoms, any atom in the overlap is part of at most two rings, and no atoms in the ring overlap with bridged rings. Finally, bridged rings are rings that share more than two atoms with another ring, contain atoms that are part of three or more rings, or share atoms with another bridged ring (Fig. 2A).

After ring systems have been identified, atoms are placed onto a 2D coordinate system. If the molecule contains rings, positioning starts with the placement of an atom in a ring, prioritising bridged rings over simple and overlapping rings. Then, the graph is traversed one atom at a time in depth-first fashion. If an atom is part of a ring, the entire ring or ring system get placed at once. In the case of simple and overlapping rings, ring placement can be done using simple polygon geometry. For bridged rings, atoms are positioned using the force-spring model described by Kamada and Kawai [9], where all atoms of the bridged system are initially placed in a circle, and then pulled towards their optimal positions by minimising the difference between the desired bond length and the distance between neighbouring atoms, and maximising distances between non-neighbouring atoms. Non-ring atoms are positioned a bond length away from the previous atom, where the angle with respect to the previous atom is determined by the number of neighbours the atom has (Fig. 2C), and the size of the molecular subtree behind each neighbouring atom (Fig. 2D). Stereochemically restricted double bonds are always forced into the appropriate cis- or trans conformation. Unlike SmilesDrawer, which directly infers bond stereochemistry from the SMILES string, PIKAChU draws this information from bond objects stored in the molecular graph. As an improvement on SmilesDrawer, PIKAChU attempts to resolve wrongly depicted stereobonds in rings by mirroring one of the neighbouring atoms into the ring. PIKAChU always selects the atom with the smallest protruding side chain for this purpose (Additional file 2: Fig. S3). When multiple consecutive stereobonds are found in a ring, PIKAChU adjusts them in order, never rotating a neighbour of the same bond twice.

Once all atoms have been assigned initial coordinates, atoms adjacent to rings are flipped outside of their ring where possible. Then, the drawing is checked for overlaps between atoms, and these overlaps are resolved by rotating branches of the molecule around single bonds. In PIKAChU, we have included an extra ‘finetuning’ option that is not present in SmilesDrawer. When the finetuning flag is set to True, all pairs of clashing atoms are detected. Then, the shortest path is calculated between all clashing atoms. First, PIKAChU determines which bonds are rotatable: bonds are considered unrotatable when they are a chiral bond, are adjacent to a chiral bond, or are in a cycle. As rotations around bonds located equally far away from two clashing atoms likely have the greatest impact on clash resolution, PIKAChU selects the rotatable bond that is positioned as close to the centre of the shortest path as possible. Next, PIKAChU takes the resulting set of bonds found for all clashes, and rotates each at 30° intervals, assessing and storing the number of clashes in the drawing after each iteration. The angle for which the number of steric clashes is minimised is chosen (Fig. 2B).

Finally, some bonds adjacent to chiral centres are replaced with backward and forward wedges. They are placed such that they do not neighbour more than one chiral centre where possible, they are not part of a ring, and point in the direction of the shortest branch leading from a chiral centre, in that order of priority. The resulting image is subsequently written to a.svg or.png file or displayed directly in matplotlib.

### Structure annotation

PIKAChU provides the option to add custom annotations to structures. Each Atom instance contains an ‘annotations’ attribute, which points to an AtomAnnotation instance. An AtomAnnotations instance can contain as many annotations as the user requires. Annotations can be added to all atoms in a structure at once by defining the name of the attribute with a string, and optionally providing a default value for the attribute. Subsequently, specific values can be added and retrieved for specific atoms or atom sets. A manual providing an example can be found on the PIKAChU wiki.

### Substructure matching

PIKAChU detects occurrences of a substructure in a superstructure in five steps. In all steps, hydrogens are ignored. First, PIKAChU checks for each atom type in the substructure if enough atoms of these types are accounted for in the superstructure. Second, it assesses for each atom in the substructure whether an atom exists in the superstructure with the same connectivity, looking at directly neighbouring bonds and atoms. Third, using the atom with the most diverse connectivity as a seed, it finds matches of the substructure in the superstructure using a depth-first search algorithm, ignoring stereochemistry. By first looking at atom type and atom connectivity, and by using atoms of diverse connectivity as seeds for substructure matching, the number of calls to the computationally expensive depth-first search function is minimised. Fourth, for each match, it determines if all chiral centres in the substructure have the same orientation as corresponding chiral centres in the superstructure. Fifth, PIKAChU checks if cis–trans orientation of double bonds in the substructure matches that of double bonds in the superstructure. Chiral centre and double bond stereochemistry checks can be toggled by the user independently of one another. If chirality of bonds and atoms are considered, substructures with undefined stereochemistry will still match to parent structures with defined stereochemistry. This does not apply in reverse: if a stereocentre or stereobond is defined for a substructure, it will not match to parent structures with undefined stereochemistry.

The algorithm described is somewhat similar to the Ullmann algorithm [19], which first assesses if a candidate subgraph contains enough nodes of the correct degree prior to substructure matching and selects nodes of the most unique degree as seeds. A key difference is that PIKAChU’s substructure matching algorithm also takes into account the identity of a node’s neighbours, not just a node’s degree.

Substructures can be easily visualised through a range of functions in PIKAChU’s ‘general’ 'library.

### Fingerprinting

PIKAChU uses ECFP [15], which is an improved version of the classical Morgan fingerprinting also taking into account cycle membership, to perform similarity searches and convert molecules to bit vectors for machine learning featurisation. Using Python’s inbuilt hashlib library, PIKAChU initialises each atom to a 32-bit hash, derived from a tuple containing information on heavy neighbours, valence, atomic number, atomic weight, charge, hydrogen neighbours, and ring membership. Then, each atom hash is iteratively updated with hashes from its neighbours, as well as the distance from the neighbour to the atom and stereochemical information if the atom is a chiral centre. The number of iterations depends on a radius which can be set to any number (default = 2 for ECFP-4 fingerprinting). The ECFP algorithm was described in detail by Rogers and Hahn in 2010 [15]. Finally, duplicate hashes are removed, as well as different hashes representing the same substructure, yielding a set of 32-bit hashes that constitutes a molecule’s fingerprint.

Using ECFP fingerprinting, PIKAChU can calculate Jaccard/Tanimoto distance and/or similarity between any two molecules. Furthermore, PIKAChU can convert molecule libraries into bit vectors of varying lengths (default = 1024) and an accompanying list of substructures represented by those bit vectors that can be used in downstream machine learning algorithms.

### Defining reaction targets

In order to facilitate implementation of reactions and reaction pathways, PIKAChU lets users define target bonds or atoms within substructures with a set of dedicated functions. These functions take a SMILES string representing a substructure, and either one or two integers that define an atom or a bond between two atoms respectively. For example, the SMILES string ‘C(= O)NC’, accompanied by the integers 0 (pointing to the first C atom) and 2 (pointing to the N atom), represents a peptide bond. The occurrences of these bonds/atoms are then detected within a superstructure through a substructure search and are returned as a list of bonds/atoms. Subsequently, the returned bonds/atoms can be used as reaction targets, for instance for bond hydrolysis or atom methylation, using functions in PIKAChU for breaking or creating bonds and adding or removing atoms. Reactions currently have to be encoded manually using a library of functions included in PIKAChU, which include functions for creating bonds, breaking bonds, adding and removing atoms, and splitting disconnected graphs into separate structures. We provided in-built condensation and hydrolysis functions, as well as a more elaborate ketoreductase function, as examples on our GitHub page.

### Characterisation and visualisation of the polyketide ketoreduction reaction

We demonstrated the implementation of reactions using PIKAChU by characterising and visualising a polyketide ketoreduction reaction. We built the ketoreduction reaction by first defining a reaction target as described above, in this case a β-keto bond, and detecting its position in a polyketide chain. Next, we wrote a function that reduces the double carbonyl bond to a single bond, which identifies and removes the π-electrons in the double bond, sets the bond type to single, adjusts the hybridisation of the atoms involved and finally updates the structure object through PIKAChU’s refresh functions. To finalize the reaction, two hydrogen atoms were added to the carbon and oxygen atoms of the former carbonyl bond using PIKAChU’s add_atom function. Finally, to visualise the reaction, we highlighted the atoms and bonds of the newly formed hydroxyl group in red and drew the molecule.

Detailed instructions on how to make full use of PIKAChU’s range of functionalities, as well as the script used to implement the ketoreduction reaction, can be found in the online documentation.

### Validation

To assess the correctness of PIKAChU’s SMILES reading and writing software, we converted all SMILES strings from the NP Atlas database into PIKAChU Structure instances. Subsequently, we converted these structure instances back to SMILES strings. Next, we canonicalized the PIKAChU-generated SMILES and the original SMILES using RDKit (v2020.09.1.0), setting the ‘isomericSmiles’ flag to ‘True’ such that correct interpretation of cis–trans bond configuration and the stereochemistry of chiral centres could also be assessed. If the two canonicalized SMILES were identical, a SMILES to structure to SMILES conversion was considered correct.

To measure PIKAChU’s drawing readability, atom coordinates were computed with PIKAChU and RDKit’s rdCoordGen module (v2020.09.1.0) for the 32,552 molecules in the NPAtlas database (v2021_08) and the 100,000 smallest molecules from the ChEMBL database (release 30). Next, all drawings were assessed for clashes. A clash was defined as two non-neighbouring atoms sitting at less than half an average bond length distance from each other in Euclidean space. Total number of clashes, number of structures containing clashes, and the number of structures that gave drawing errors were recorded.

To assess PIKAChU’s drawing accuracy, we included a MOL file writer into PIKAChU, which stores PIKAChU-computed atom coordinates and connectivities as a MOL file. We generated such MOL files for the entire NP Atlas database and the 100,000 smallest molecules from the ChEMBL database, read the resulting MOL files with RDKit’s rdMolFiles module (v2020.09.1.0), stored the resulting molecules as SMILES strings, and using RDKit canonicalized both the original input SMILES and the SMILES produced from the PIKAChU-generated MOL files setting the ‘isomericSmiles’ flag to ‘True’. If the SMILES were identical, a PIKAChU-generated drawing was considered ‘correct’.

### Speed assessment

PIKAChU’s speed was assessed with Python’s ‘time’ module. As we particularly designed PIKAChU with natural product chemistry in mind, which typically involves larger and more heavily cyclised compounds than most molecules stored in small-molecule databases, we decided to test drawing speed on two different databases: the NP Atlas database and the ChEMBL database. For each database, we randomly selected 10,000 molecules and timed drawing speed at 10, 20, 50, 100, 200, 500, 1000, 2000, 5000 and 10,000 drawn structures using Python’s ‘time’ module.

## Results and discussion

PIKAChU is a dependency-light cheminformatics kit implemented entirely in Python. With only matplotlib as dependency and an extensive readme, wiki, tutorials, and example scripts on its GitHub page, PIKAChU is easy to run and install, and suitable for integration into bioinformatics and cheminformatics pipelines. Below, we will first assess PIKAChU’s ability to correctly interpret SMILES, draw structures accurately and readably, detect and visualize substructures, and perform ECFP fingerprinting. We also measured PIKAChU’s SMILES reading and structure drawing speed. Next, we demonstrate how PIKAChU can be used to implement and visualise reactions. Finally, we compare PIKAChU to the state-of-the-art cheminformatics kits/chemical drawing libraries RDKit, ChemDraw and SmilesDrawer.

### SMILES reading and writing

We assessed PIKAChU’s ability to parse and generate correct SMILES syntax by comparing the SMILES it converts to SMILES generated by RDKit, a well-established cheminformatics package. As PIKAChU was created with natural product chemistry in mind, which typically involves large and heavily cyclised molecules the function of which depends heavily on stereochemistry, we performed our validation with the NP Atlas database. This database contains 32,552 manually curated natural product structures and their corresponding isomeric SMILES strings. PIKAChU failed to convert 1 SMILES from NP Atlas (~ 0.003%) to structure graphs, which was an erroneous SMILES describing a nitrogen atom with a valency of 5, which is impossible considering that nitrogen only has four electron orbitals available for bonding in its valence shell. This SMILES attempted to describe a nitro group, which PIKAChU tolerates even for valency 5 nitrogens. However, this representation of a nitro group was unconventional and incorrect, with two superfluous hydrogen atoms attached to the nitrogen. This demonstrates how the detailed graph-based, object-oriented encoding of chemical structures down to the electron level in PIKACHU intrinsically ensures that all structures that are loaded are chemically valid. Of the remaining 32,551 SMILES-to-structure-graph-to-SMILES conversions, only one yielded a SMILES string that described different chemistry than the original: three carbon-13 atoms were interpreted as carbon-12 (Additional file 2: Table S1, third row). As PIKAChU does not yet support isotopic differentiation, this is not unexpected.

**Fig. 3:**
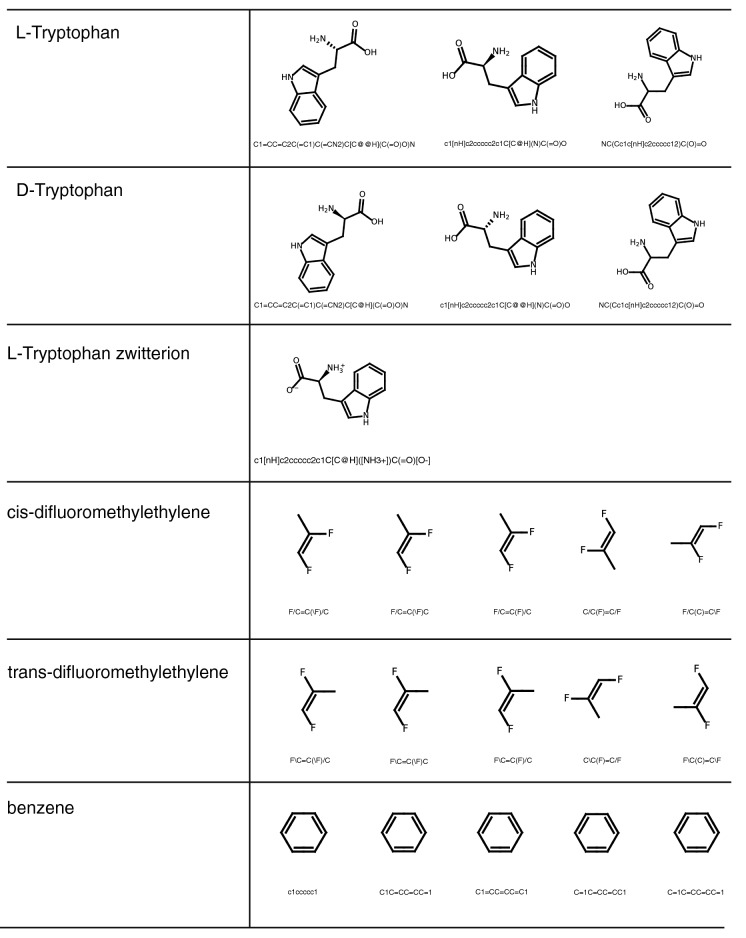
Assessment of PIKAChU's SMILES reader. Structures were drawn from the SMILES written beneath the molecule depictions. All 22 structures were correctly drawn.

Additionally, we manually assessed the correctness of 22 SMILES-to-graph conversions by reading in and subsequently drawing the SMILES in PIKAChU. We chose the SMILES such that a variety of syntax representations and chemistries were represented, including rings, aromatic systems, charge, stereocentres and bond stereochemistry. Some SMILES describe the same structures but use a different syntax. PIKAChU handled all SMILES correctly, accurately detecting and visualising all aforementioned chemical properties (Fig. 3).

PIKAChU is not suitable for reading in molecules with a high number of recursive cycles, such as buckminsterfullerene. As PIKAChU detects all possible cycles within a molecule to determine aromaticity of cyclic systems, this step takes so long to compute that the program appears to get ‘stuck’. However, there exist only a handful of examples of such molecules, none of which have any real practical biological or chemical relevance.

### Structure visualisation

Another key feature of PIKAChU is molecular visualisation from SMILES. PIKAChU’s drawing software relies on similar logic to that of SmilesDrawer, a JavaScript SMILES drawing library. In our software, we added a few improvements: we fixed cis–trans stereochemistry detection (Additional file 2: Fig. S3), included an extra overlap resolution step (Fig. 2B), and implemented an improved version for finding the smallest subset of smallest rings, a key step in correctly depicting cycles. There is always a bit of debate regarding the visualisation of molecular macrocycles. Many organic chemists opt for a ‘honeycomb’ architecture, as employed by ChemDraw and CDK, to better represent the 3D architecture of a molecule, hinting at long-distance interactions that may take place within the compound (Additional file 2: Fig. S4B). However, this representation does not instantly draw the eye to sites of cyclisation, a drawback for natural product biologists and bioinformaticians who are often interested in the biosynthetic steps involved in a compound’s assembly. As PIKAChU was created with natural product chemistry in mind, we chose to use a polygon representation for macrocycles, which clearly shows cyclisation sites (Additional file 2: Fig. S4A).

While PIKAChU always detects and interprets aromaticity internally, it currently only supports drawing structures in a kekulised format.

**Fig. 4:**
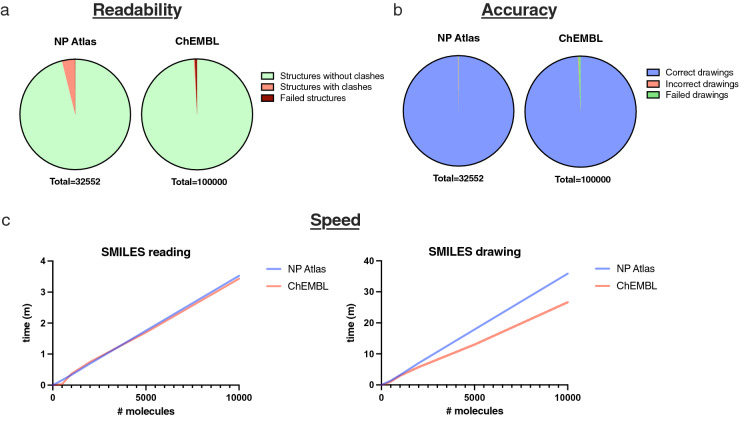
PIKAChU’s performance tested on the NP Atlas and ChEMBL databases. For drawing readability and accuracy assessment, the entire NPAtlas database and the 100,000 smallest molecules of the ChEMBL database were tested. For speed assessment, 10,000 random molecules from each database were used. **A** Structure readability expressed as the percentage of molecules with steric clashes. **B** Drawing accuracy expressed as the percentage of drawings correctly interpreted by RDKit upon writing PIKAChU-calculated atom coordinates to a .mol file. **C** PIKAChU’s SMILES reading speed and drawing speed in molecules per minute.

The most important aspect of automated molecular visualisation is accuracy: users need to be able to rely on the correctness of drawing software, especially when processing a large number of structures at once making it impossible to inspect each image independently. To this purpose, we visualised a chemically diverse set of structures from the ChEMBL and NP Atlas databases, and tested if RDKit could interpret correct chemistry from PIKAChU-generated atom coordinates. Out of the 32,552 structures in the NP Atlas database, only 40 (~ 0.12%) were drawn incorrectly. Of these, 33 were drawn wrongly due to incorrect depiction of cis–trans chemistry of double bonds adjacent to nested rings (Additional file 2: Table S1). Additionally, PIKAChU failed to convert 8 structures to drawings (~ 0.02%). One of these was the same incorrectly defined SMILES describing the valency 5 nitrogen that was found previously. The remaining failures largely resulted from ChiralityErrors: errors raised by PIKAChU when it cannot correctly depict cis/trans chemistry of a double bond. With 32,504 correctly drawn structures, PIKAChU achieves a drawing accuracy of 99.85%. PIKAChU performed comparably on the 100,000 smallest molecules from the ChEMBL database (99.21% accuracy, 0.16% incorrect drawings, 0.63% unsuccessful conversions; Fig. 4A). Comprehensive lists and example depictions of SMILES leading to incorrect interpretations can be found in Additional file 2: Table S1 and Additional file 1.

We additionally assessed the readability of these PIKAChU-rendered drawings by automatically detecting steric clashes from PIKAChU-generated atom coordinate sets. Only 4.95% of NP Atlas SMILES renderings contained steric clashes, with ~ 1.62 clashes per clashing structure. PIKAChU was better at processing the ChEMBL database, with only ~ 0.30% of drawings containing clashing atoms. This makes sense, as NP Atlas contains a higher proportion of highly cyclised systems and large molecules, properties which make it more difficult to readably depict a molecule in a plane. As the honeycomb approach of depicting molecules solves some of these issues, we hope to implement the option to visualise molecules using either the honeycomb or the polygon strategy in the future.

**Fig. 5:**
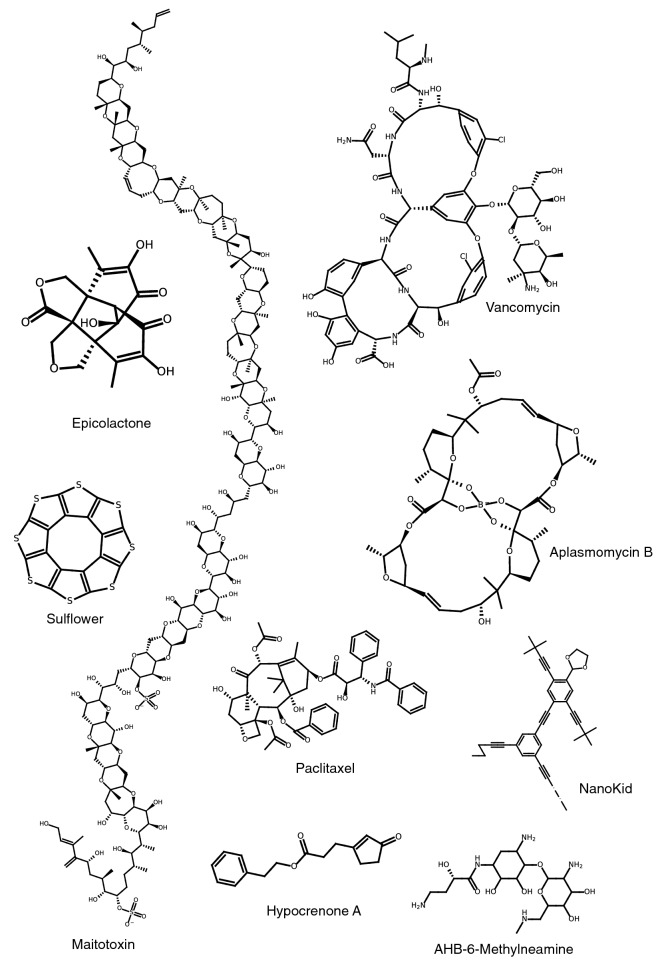
Various molecules rendered by PIKAChU

In Fig. 5, we show nine examples of structures rendered by PIKAChU. Due to the drawing algorithm that PIKAChU employs for complex ring systems, 5-membered and 6-membered rings often appear distorted, as observed for vancomycin and aplasmomycin B. Additionally, PIKAChU’s overlap resolution step, while resolving a lot of steric clashes, sometimes results in carbon–carbon bonds being placed at an 180° angle which makes the structure less interpretable, as seen for PIKAChU’s depiction of the molecule nanokid.

### Speed assessment

We assessed PIKAChU’s SMILES reading and structure drawing speed by drawing 10,000 random molecules from the NP Atlas database and the ChEMBL database (Fig. 4C). With an average reading speed of ~ 2,874 SMILES per minute and an average drawing speed of ~ 279 molecules per minute for the NP Atlas database and ~ 375 molecules per minute for the ChEMBL database on a single laptop core, it is clear that PIKAChU spends the bulk of its time rendering an image on atom positioning, not on SMILES reading. The discrepancy between the two databases can be explained by the nature of the molecules contained within them: typically, natural products are larger and more cyclised than the average small molecule. This makes PIKAChU’s drawing speed one order of magnitude slower than RDKit’s (Additional file 2: Table S2), which is expected considering that PIKAChU is a pure Python package while RDKit generates drawings with pre-compiled C++ code. Also, PIKAChU’s finetuning step is computationally expensive, likely leading to an increase in computational time. Still, PIKAChU is fast enough for integration into pure-Python bioinformatics and cheminformatics pipelines.

### Substructure detection

**Fig. 6:**
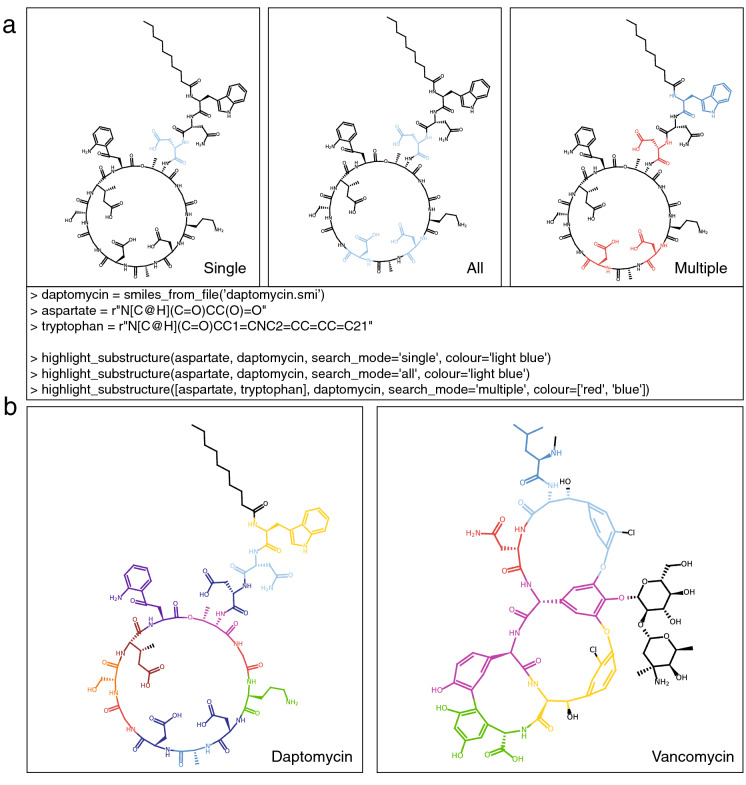
Substructure matching with PIKAChU. **A** From left to right: examples of highlighting a single instance of a substructure, all instances of a substructure, or all instances of multiple substructures. In the example, occurrences of aspartic acid and tryptophan were searched in the superstructure daptomycin. The code used to generate the images is displayed underneath the panels. **B** PIKAChU’s substructure matching algorithm using to visualise all amino acid components of the antibiotics daptomycin (left) and vancomycin (right). Code can be found at https://github.com/BTheDragonMaster/pikachu/blob/main/example_scripts/amino_acid_composition.py

A dedicated set of functions ensures that performing substructure searches using PIKAChU is straightforward. With a single line of code, users can visualise a single occurrence of a substructure, all occurrences of a substructure, or all occurrences of a range of substructures in a chemical compound (Fig. 6A). Substructure searches are fast due to several pre-processing steps, ensuring that the expensive graph matching algorithm is only executed when a match is likely. Stereochemistry matching, activated by default, can be toggled on and off.

With PIKAChU’s substructure matching algorithm, we visualised the amino acid composition of the cyclic peptides daptomycin and vancomycin, using only a single line of code for each (Fig. 6B). Colours are fully and easily customisable, and can be provided as hex codes or as colour names.

### ECFP fingerprinting

To quickly determine the approximate similarity between two molecules, PIKAChU employs ECFP fingerprinting [15]. PIKAChU hashes each molecule into a set of unique identifiers, each of which represents a substructure. Collectively, these identifiers make up a molecule’s fingerprint. Then, PIKAChU calculates the Jaccard/Tanimoto similarity between two molecules by comparing their fingerprints, giving a measure of molecular similarity and/or distance.

Here, we showcase PIKAChU’s ECFP fingerprinting by calculating and subsequently constructing a tSNE plot of the molecular distances between 36 calcium-dependent lipopeptides. Lipopeptides of the same family grouped together (Additional file 2: Fig. S5), confirming that PIKAChU’s ECFP fingerprinting yields reliable measures of chemical similarity.

Additionally, PIKAChU’s ECFP fingerprinting makes it possible to generate bit vectors from molecule sets, where each element in the vector represents the presence/absence of a specific substructure. These can subsequently be used as interpretable molecular featurisations for machine learning.

### Building in silico reactions using PIKAChU

PIKAChU provides a platform for the creation and visualisation of reaction mechanisms by providing a range of reaction functions that can be used to make or break molecular bonds, add or remove atoms and alter the chirality of stereocentres. In addition to these built-in reaction building blocks, PIKAChU allows users to easily define more complex reactions through the manipulation of atom and bond object attributes. Additionally, PIKAChU supports fully customisable structure annotation, which is useful for keeping track of reaction steps, reaction targets, or atom origin. As a proof of principle, we used PIKAChU to define and visualise a polyketide ketoreduction reaction, catalysed by a ketoreductase polyketide synthase domain during polyketide synthesis, employing both built-in and custom reaction functions (Additional file 2: Fig. S6). This example, as well as a comprehensive guide containing instructions on how to build reaction mechanisms using PIKAChU, can be found in the online documentation.

While creating reaction pathways with PIKAChU enforces chemically correct conversions by checking at each step if a structure is chemically correct, it is more laborious than similar functionalities in other cheminformatics kits such as RDKit, which use reaction SMILES and atom mapping to perform chemical reactions. We intend to implement reaction SMILES and atom mapping into PIKAChU in the future.

### PIKAChU compared to state-of-the-art chemical drawing software

**Fig. 7:**
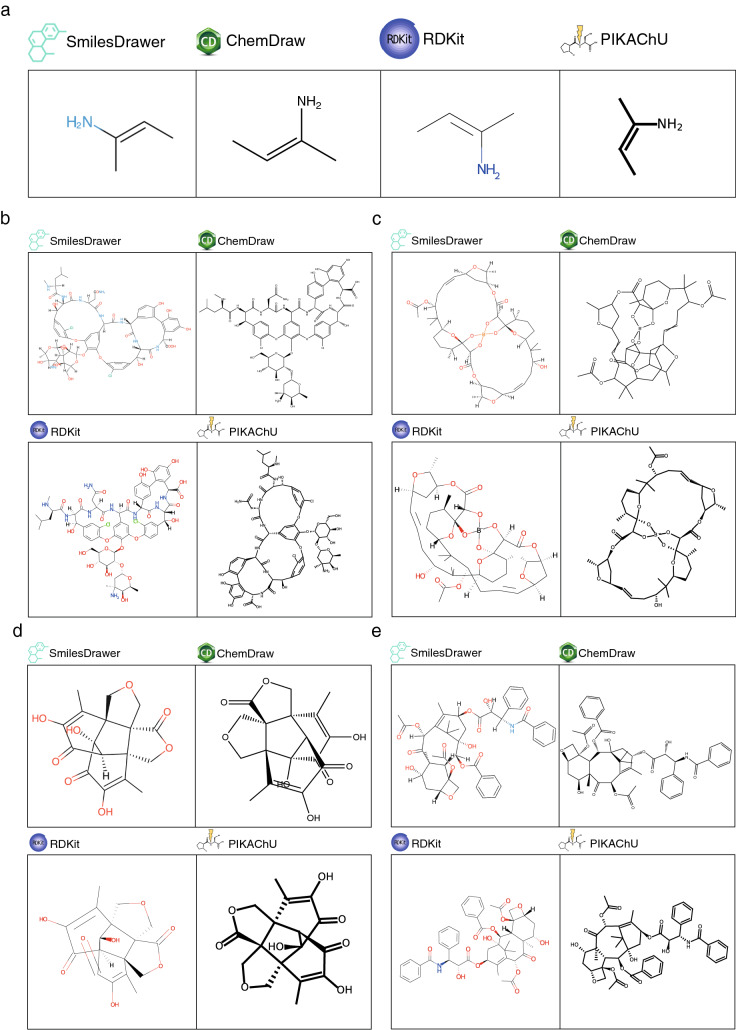
Comparison of PIKAChU to various other chemical drawing software packages. **A** SmilesDrawer, ChemDraw, RDKit and PIKAChU drawings given the SMILES string ‘C/C=C(\N)/C’. While ChemDraw, RDKit and PIKAChU all draw the cis–trans stereochemistry of the double bond correctly, with the amino group cis of the methyl group, SmilesDrawer draws the stereobond in the wrong orientation. **B** SmilesDrawer, ChemDraw, RDKit and PIKAChU drawings of the heavily cyclised molecule vancomycin. **C** SmilesDrawer, ChemDraw, RDKit and PIKAChU drawings of the molecule aplasmomycin B, **D** epicolactone, and **E** paclitaxel.

Finally, we assessed how PIKAChU performs compared to existing chemical drawing software. To this purpose, we visualised various structures in PIKAChU (v1.0.5), RDKit (v2020.09.1.0), ChemDraw (v20.1.0.112) and SmilesDrawer (v1,2.0), and manually assessed drawing quality and correctness (Fig. 7). Only SmilesDrawer occasionally produced an incorrect structure, confusing cis–trans stereochemistry when stereochemistry is defined in or after a branch (Fig. 7A). For heavily cyclised molecules (Fig. 7B–E), we see a difference between the ‘honeycomb’ (RDKit and ChemDraw) and the ‘polygon’ (PIKAChU and SmilesDrawer) approaches of cycle positioning. The honeycomb approach ensures minimal distortion of microcycles, even when they are part of larger systems; as such, RDKit and ChemDraw render molecules such as vancomycin with fewer distortions than SmilesDrawer and PIKAChU (Fig. 7B). However, when the honeycomb approach does not work because of steric constraints, forcing microcycles into regular polygons can distort the macrocyclic structure to the extent that the drawing becomes unreadable. This is the case for aplasmomycin B (Fig. 7C), which is drawn with fewer bond overlaps by SmilesDrawer and PIKAChU. In structures where microcycles and macrocycles are separate, there is little difference in structure rendering between the two approaches (Fig. 7D, E). It must be said that while readable, these depictions are still far from optimal, and chemists could produce better diagrams by manually tweaking the drawings of these molecules in ChemDraw. This demonstrates that even with state-of-the-art software, 2-dimensional automatic visualisation remains a challenge, particularly for constrained ring systems.

PIKAChU has a slight advantage over RDKit in drawing molecules of varying sizes, automatically adjusting the canvas size based on the size of the molecule to be drawn. This also means that PIKAChU’s font size, bond length and bond thickness maintain a constant ratio across different drawings, which is not the case for RDKit (Fig. 7D, E). While it is possible to manually adjust canvas size in RDKit, some extra coding steps are required to achieve this.

PIKAChU’s visual output is far more customizable than that of SmilesDrawer, allowing for molecule rotation, drawing multiple molecules on a single canvas, and custom colouring of each individual bond and atom, supporting hex-codes as well as a range of descriptive strings.

While ChemDraw accommodates high-quality and highly customisable visualisation, it is not open-source. This makes PIKAChU more suitable for integration into automated open-source pipelines required by many projects.

## Conclusions

We developed PIKAChU, a dependency-light cheminformatics library implemented entirely in Python. Having extensively validated our software, we conclude that, while RDKit heavily outperforms PIKAChU in terms of speed, PIKAChU performs sufficiently fast and reliably to be suitable for cheminformatics and bioinformatics pipelines. Backed by extensive online documentation, easy and straightforward installation, and state-of-the-art automated visualisation software, we hope that PIKAChU can provide a convenient alternative for chem- and bioinformaticians programming in Python.

## Supplementary Information

**Additional file 1: **True failed SMILES. SMILES failed because of incorrect chemistry.

**Additional file 2: Figure S1.** PIKAChU’s recursive aromaticity detection. Aromatic cycles are individually and recursively detected and later joined into cyclic systems. **Figure S2.** Faulty ring detection by SmilesDrawer leads to unreadable structure renderings. SmilesDrawer’s SSSR implementation fails to detect one of the macrocycles, leading to the unreadable structure shown on the right. PIKAChU’s SSSR implementation (left) does recognise this ring, and therefore renders the structure correctly. **Figure S3.** PIKAChU resolves incorrectly drawn chiral bonds in rings. PIKAChU (left) correctly depicts cis-trans chemistry of stereobonds in rings that SmilesDrawer (right) cannot visualise. **Figure S4.** Two approaches for visualising macrocycles. A. Daptomycin visualised using the 'polygon' approach in PIKAChU. B. Daptomycin visualised using the 'honeycomb’ approach in ChemDraw. **Figure S5.** tSNE plot of 36 calcium-dependent lipopeptides drawn in a 2D plane based on the Tanimoto distances between their structures as computed by PIKAChU. The script and structures used to draw this figure can be found in the example_scripts and example_structures folders in GitHub, respectively. **Figure S6.** Visualisation of the polyketide ketoreduction reaction, built and visualised by PIKAChU. The reduced group is highlighted in red. The script performing this reaction can be found on GitHub. Supplementary **Table S1.** Examples of SMILES that PIKAChU does not draw correctly, compared to ChemDraw drawings drawn from the same SMILES. **Table S2.** Drawing times of molecules rendered by RDKit and PIKAChU. Time is indicated in minutes.

## Data Availability

The PIKAChU software is made available under an open-source (MIT) license and can be found at https://github.com/BTheDragonMaster/pikachu. A wiki can be found at https://github.com/BTheDragonMaster/pikachu/wiki. Scripts used for the results section of this paper are made available at https://github.com/BTheDragonMaster/pikachu/tree/main/example_scripts. The NPAtlas database used in our analyses can be downloaded at https://www.npatlas.org/download.

## Abbreviations

PIKAChU: Python-based Informatics Kit for Analysing Chemical Units
SMILES: Simplified Molecular-Input Line Entry System
ECFP: Extended Connectivity FingerPrinting
