# Supplementary material for: PIKAChU: a Python-based informatics kit for analysing chemical units
Source: J Cheminform. 2022 Jun 7;14:34. doi: 10.1186/s13321-022-00616-5 (PMC9172152; doi:10.1186/s13321-022-00616-5)
Supplement: Supplementary file 2 — Additional file 2: Figure S1. PIKAChU’s recursive aromaticity detection. Aromatic cycles are individually and recursively detected and later joined into cyclic systems. Figure S2. Faulty ring detection by SmilesDrawer leads to unreadable structure renderings. SmilesDrawer’s SSSR implementation fails to detect one of the macrocycles, leading to the unreadable structure shown on the right. PIKAChU’s SSSR implementation (left) does recognise this ring, and therefore renders the structure correctly. Figure S3. PIKAChU resolves incorrectly drawn chiral bonds in rings. PIKAChU (left) correctly depicts cis-trans chemistry of stereobonds in rings that SmilesDrawer (right) cannot visualise. Figure S4. Two approaches for visualising macrocycles. A. Daptomycin visualised using the 'polygon' approach in PIKAChU. B. Daptomycin visualised using the 'honeycomb’ approach in ChemDraw. Figure S5. tSNE plot of 36 calcium-dependent lipopeptides drawn in a 2D plane based on the Tanimoto distances between their structures as computed by PIKAChU. The script and structures used to draw this figure can be found in the example_scripts and example_structures folders in GitHub, respectively. Figure S6. Visualisation of the polyketide ketoreduction reaction, built and visualised by PIKAChU. The reduced group is highlighted in red. The script performing this reaction can be found on GitHub. Supplementary Table S1. Examples of SMILES that PIKAChU does not draw correctly, compared to ChemDraw drawings drawn from the same SMILES. Table S2. Drawing times of molecules rendered by RDKit and PIKAChU. Time is indicated in minutes. [file 13321_2022_616_MOESM2_ESM.docx]

**FIGURES**

**
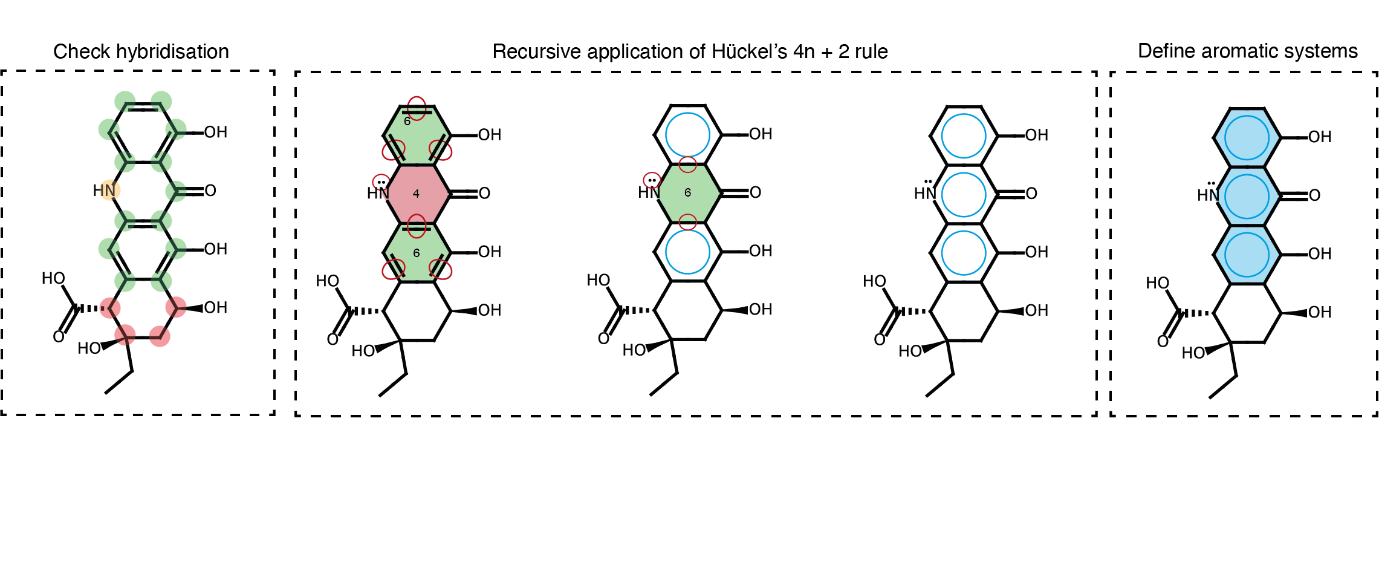
**

***Figure S1. PIKAChU’s recursive aromaticity detection.*** *Aromatic cycles are individually and recursively detected and later joined into cyclic systems.*

*
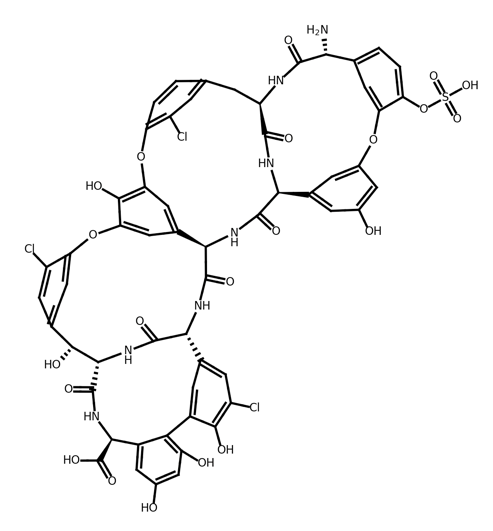

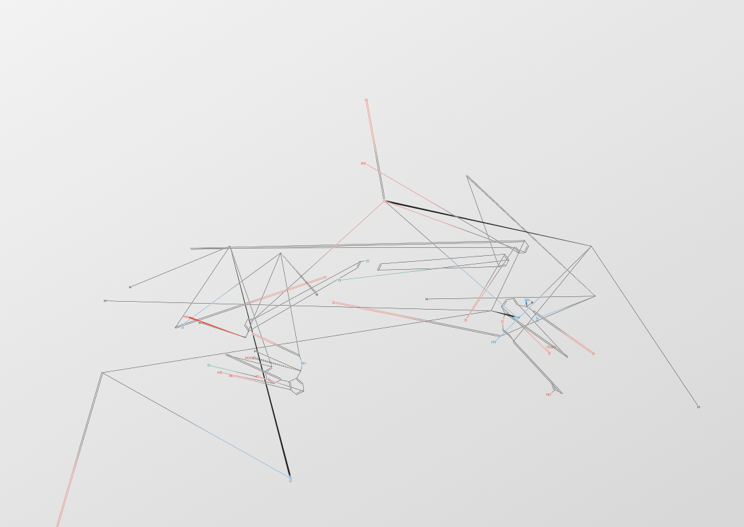
*

*SMILES:*

*N[C@@H]1C2=CC=C(OS(O)(=O)=O)C(OC3=CC(=CC(O)=C3)[C@@H]3NC(=O)[C@@H](CC4=CC(Cl)=C(OC5=C(O)C6=CC(=C5)[C@@H](NC3=O)C(=O)N[C@@H]3C5=CC(Cl)=C(O)C(=C5)C5=C(O)C=C(O)C=C5[C@H](NC(=O)[C@@H](NC3=O)[C@H](O)C3=CC=C(O6)C(Cl)=C3)C(O)=O)C=C4)NC1=O)=C2*

***Figure S2. Faulty ring detection by SmilesDrawer leads to unreadable structure renderings.*** *SmilesDrawer’s SSSR implementation fails to detect one of the macrocycles, leading to the unreadable structure shown on the right. PIKAChU’s SSSR implementation (left) does recognise this ring, and therefore renders the structure correctly.*

*
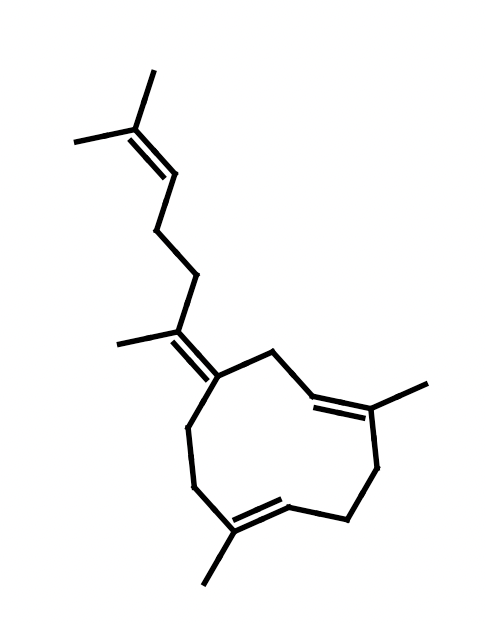

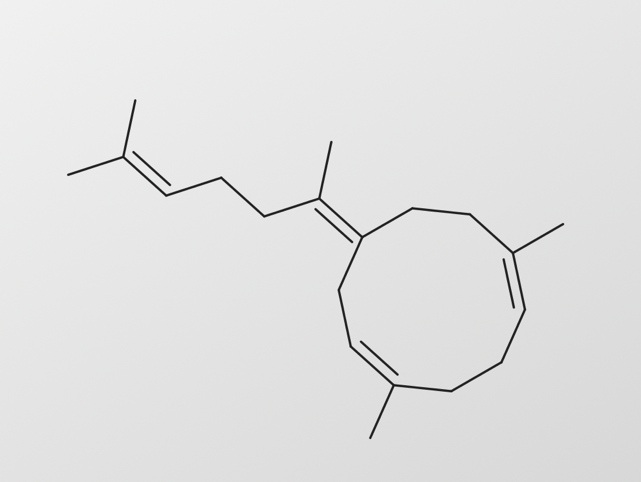
*

*SMILES: C/C/1=C\CC/C(=C/CC(=C(C)CCC=C(C)C)CC1)/C*

***Figure S3. PIKAChU resolves incorrectly drawn chiral bonds in rings.*** *PIKAChU (left) correctly depicts cis-trans chemistry of stereobonds in rings that SmilesDrawer (right) cannot visualise.*


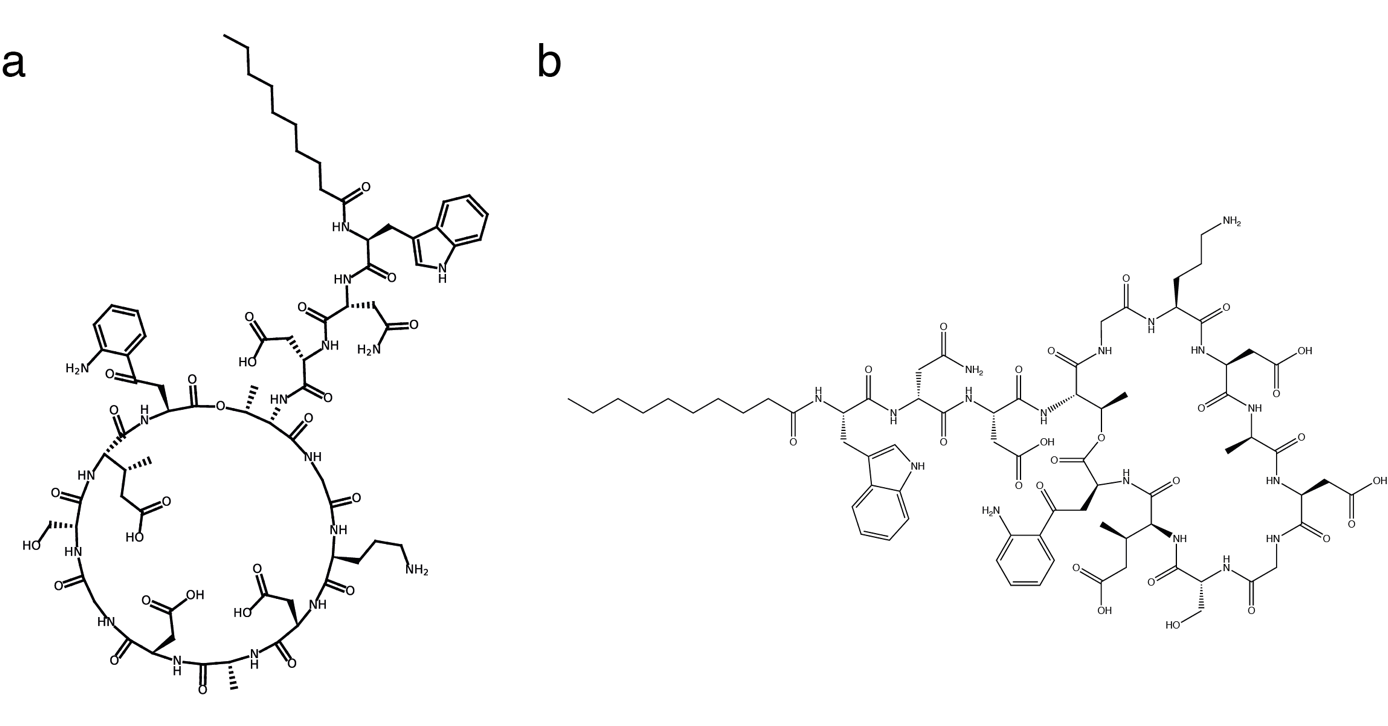


**Figure S4. Two approaches for visualising macrocycles.** A. Daptomycin visualised using the 'polygon' approach in PIKAChU. B. Daptomycin visualised using the 'honeycomb’ approach in ChemDraw.


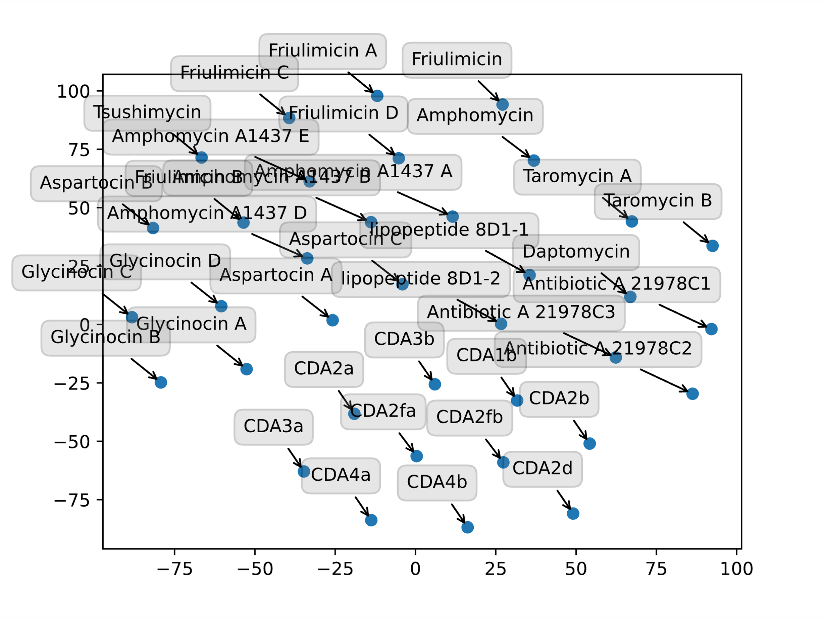


**Figure S5: tSNE plot of 36 calcium-dependent lipopeptides drawn in a 2D plane based on the Tanimoto distances between their structures as computed by PIKAChU.** The script and structures used to draw this figure can be found in the example_scripts and example_structures folders in GitHub, respectively.

*
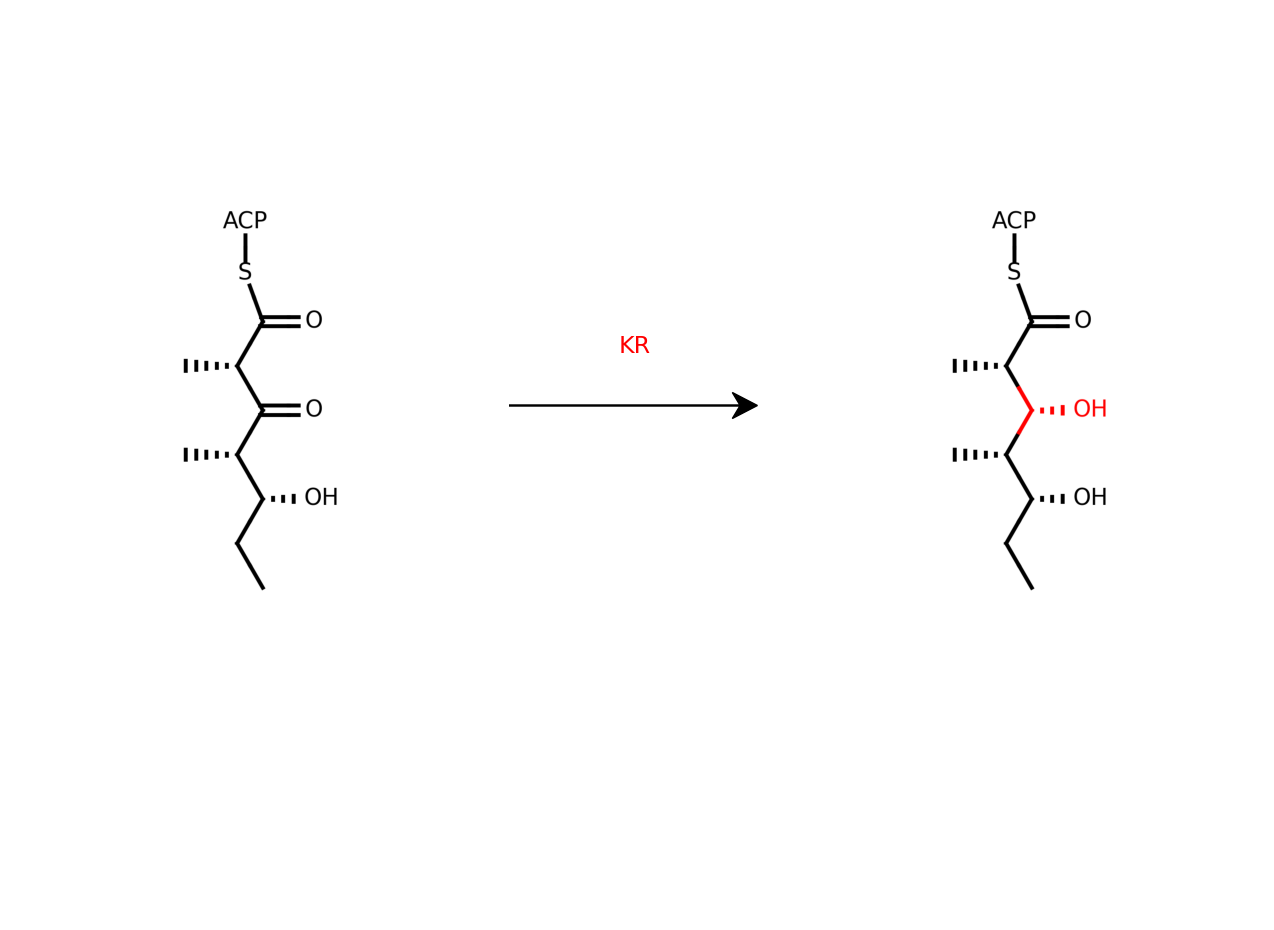
*

***Figure S6. Visualisation of the polyketide ketoreduction reaction, built and visualised by PIKAChU.*** *The reduced group is highlighted in red. The script performing this reaction can be found on* [*GitHub*](https://github.com/BTheDragonMaster/pikachu/blob/main/example_scripts/ketoreductase.py)*.*

***Table S1. Examples of SMILES that PIKAChU does not draw correctly, compared to ChemDraw drawings drawn from the same SMILES.*** *Incorrect drawings can be grouped into four groups. The most prevalent group (33 out of 39 members) comprises structures where a double bond was drawn adjacent to two rings – PIKAChU cannot yet reliably draw these structures. The last structure was correctly drawn by PIKAChU when compared to the ChemDraw drawings, but RDKit did not interpret the two stereocentres correctly. SMILES strings of all group members can be found in supplementary_file_2.xlsx*

| **PIKAChU** | **ChemDraw** |
| --- | --- |
| Double bond adjacent to two rings (33 structures) | |
| *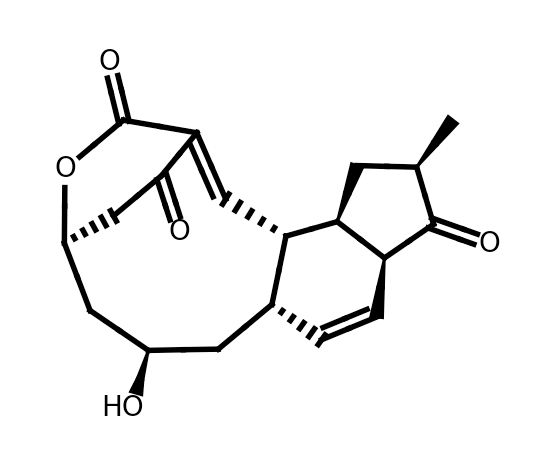* | ** |
| Cis/trans bonds flipped after application of K&K layout algorithm (4 structures) | |
| 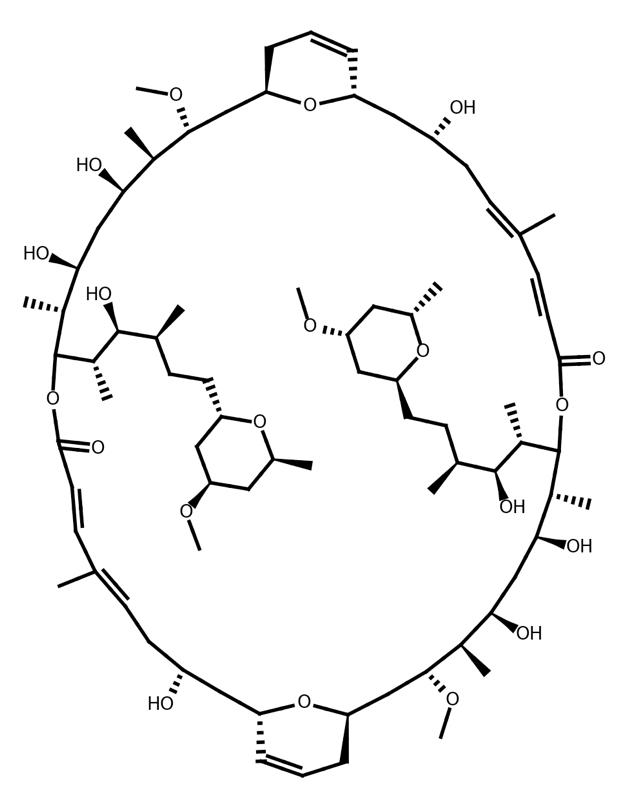 |  |
| Isotopic mass definition (1 structure) | |
| 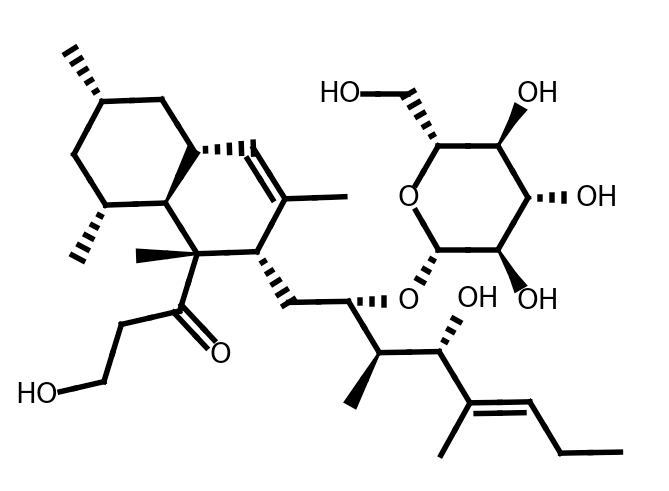 |  |
| Chiral centre (1 structure), misinterpreted by RDKit | |
| 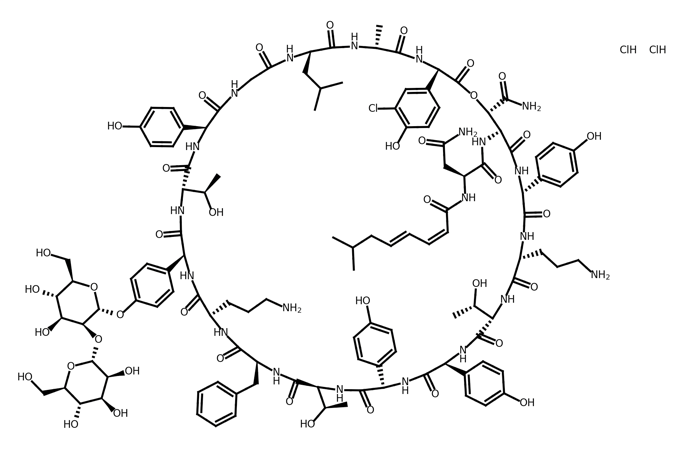 |  |

***Table S2. Drawing times of molecules rendered by RDKit and PIKAChU.*** *Time is indicated in minutes.*

| # of molecules | PIKAChU, NP Atlas | PIKAChU, ChemBL | RDKit, NP Atlas | RDKit, ChemBL |
| --- | --- | --- | --- | --- |
| 10 | 0.051 | 0.011 | 0.005 | 0.001 |
| 20 | 0.079 | 0.023 | 0.008 | 0.002 |
| 50 | 0.142 | 0.056 | 0.016 | 0.005 |
| 100 | 0.292 | 0.173 | 0.039 | 0.018 |
| 200 | 0.559 | 0.317 | 0.070 | 0.033 |
| 500 | 1.382 | 1.078 | 0.173 | 0.095 |
| 1000 | 3.178 | 2.976 | 0.370 | 0.245 |
| 2000 | 7.061 | 5.750 | 0.759 | 0.416 |
| 5000 | 17.874 | 13.031 | 1.944 | 0.975 |
| 10000 | 35.878 | 26.638 | 3.956 | 1.994 |
